# Relationship matters: a qualitative study of medical students' experiences in a learner-driven research program in South Korea

**DOI:** 10.1186/s12909-023-04337-7

**Published:** 2023-05-16

**Authors:** Hyo Jeong Lee, Ye Ji Kang, Seung-Hee Lee, Yanyan Lin, Do-Hwan Kim, Jungjoon Ihm

**Affiliations:** 1 Department of Medical Education, College of Medicine, Hanyang University, 222 Wangsimni-Ro, Seongdong-Gu, Seoul, 04763 Republic of Korea; 2 Department of Medical Education, College of Medicine, Seoul National University, Seoul, Korea; 3 Dental Research Institute, School of Dentistry, Seoul National University, 1, Gwanak-Ro, Gwanak-Gu, Seoul, 08826 Republic of Korea

**Keywords:** Research activities, Student research engagement, Research competencies, Medical student, Interpersonal relation, South Korea

## Abstract

**Background:**

Although research experience is important for medical students, it is difficult to develop research skills only through a formal curriculum. To develop research programs that address the authentic needs of students and align with the entirety of the medical school curriculum, a learner-centered approach may be more effective than an instructor-centered approach. This study investigates medical student perspectives on factors that help them develop research competency.

**Methods:**

Hanyang University College of Medicine in South Korea operates the Medical Scientist Training Program (MSTP) as a supplement to its formal curriculum. Semi-structured interviews were held with 18 students (20 cases) in the program, and qualitative content analysis was performed using the software tool MAXQDA20.

**Results:**

The findings are discussed in relation to three domains: learner engagement, instructional design, and program development. The students were more engaged when they perceived the program as a new experience, had prior research experience, wanted to make a good impression, and felt a sense of contribution. In terms of instructional design, they positively participated in research when their supervisors respected them, set clear tasks, provided constructive feedback, and invited them into the research community. In particular, the students highly valued relationships with their professors, and these relationships served not only as a main motivating factor in their research participation but also affected their college lives and careers.

**Conclusions:**

The longitudinal relationship between students and professors has newly emerged in the Korean context as a factor that strengthens student engagement in research and the complementary relationship between formal curriculum and MSTP was highlighted to encourage student engagement in research.

## Background

Research skills are a key competency for doctors who need to keep knowledge current in the ever-changing medical field. To help students develop these skills, medical schools striveto provide a variety of research opportunities, including mandatory, elective, and optional programs [1]. Participating in research prior to graduation helps medical students integrate scientific and clinical knowledge, thereby improving the quality of patient care at clinical sites after graduation [2]. While research experience is important for all medical students, it is especially important for those wanting to become physician scientists. However, in Korea, the number of physician scientists is low, with very few students choosing to practice basic medicine after graduating from medical school, and the number of clinicians attempting research and clinical trials continues to decrease [3, 4]. Medical education in Korea focuses on patient treatment, and students are concerned about a poor research environment, limited research funding, and a lack of opportunities to conduct research if they pursue basic medicine [4].

Many previous works have investigated the educational outcomes of medical research programs. For instance, several studies found that research experience increases students’ interest in research and fosters research-related competencies such as information collection, analytical skills, critical appraisal, and inference-making ability [5–7]. Moreover, through research programs, students develop scientific writing skills, the ability to accept feedback, and skills to work with team members, which are important for building their research-related competencies [8–10]. They not only increase their curiosity in research but also receive help in choosing their majors and contribute to future career development [11]. Research programs ensure that medical students are engaged in research and academic jobs, which in turn raises the completion rate of the degree program and strengthens research in the medical field [12–14].

However, irrespective of whether they complete a research program or not, some students lose interest in research or fail in their confidence when faced with challenges [11, 15, 16]. For instance, students who lack knowledge related to research and are passively involved may complain about psychological difficulties throughout the program duration [11, 17]. These students are less motivated to perform research or participate in follow-up studies as they do not plan to pursue a career in research [18, 19]. Finally, the research activities may prove excessively challenging, making it difficult to achieve the program’s goal of training students to be future researchers.

To identify more effective approaches to medical students’ engagement in research, previous studies have taken a qualitative approach to understanding the perspectives of students, educators, and administrators, with emphasis on the instructor viewpoint [1, 10, 20]. Most studies emphasized the role of the instructor in achieving successful outcomes. For instance, it has been reported that student participation relies greatly on the instructor's prior supervision experience and research capabilities [6, 8, 9]. Conversely, a lack of dedicated time or poor quality supervision from the instructor can have negative effects on student participation [21]. In addition, the availability of funding, research equipment, and compensation also influence program success [15, 17, 18, 22]. While the instructor-centered perspective is valuable, it also has limitations. Instructors guide students based on their preferences and biases and cannot directly observe every student's research process. This can lead to a potential discordance between students' perceptions of promoting research engagement and the instructor's perspective.

Therefore, it is crucial to explore students' roles and perspectives on research engagement. This study examines the perspectives of students enrolled in an extracurricular research-intensive program, given the challenges of developing research skills in a short period. While previous studies have utilized surveys to enhance research programs within a defined formal curriculum [6, 11], this study explores student viewpoints using a qualitative approach. It aims to understand how to strengthen research competency from a learner-centered perspective beyond the boundaries of the formal curriculum.

In the Medical Scientist Training Program (MSTP) at Hanyang University College of Medicine (HYUCM), students can participate in research independent of the time or subject matter of the regular curriculum. Students who join MSTP are motivated from the beginning because they participate voluntarily, so they can conduct advanced research with genuine interest. Their thoughts may shed light on the context in which student participation in research is promoted and what type of supports are needed. Gaining insight into students' perspectives can provide valuable information on their authentic needs and the types of support necessary to promote research engagement in alignment with the entire curriculum context [23]. This understanding is significant in ensuring the long-term effectiveness of the program and encouraging the development of student-centered research programs.

The current trend in medical education is to shift from a lecturer- to a learner-centered paradigm, and students can play a role in advocating and promoting change as catalysts for improving the curriculum [24]. Hence, this study explores factors that facilitate students’ research engagement from learner-centered narratives. The main research question is “How is student research engagement facilitated during the MSTP?” The results can also serve as basic data for the development of extracurricular research and educational programs for learners and supervisors.

## Methods

### Study context

This qualitative study was conducted at HYUCM in South Korea. The formal curriculum of Korean medical schools consists of two years of the pre-medical phase, where students learn the basic curriculum, and four years of the medical phase, which includes the advanced curriculum. The pre-medical course aims at developing students’ professional identity and teaching fundamental medical knowledge including humanities and basic sciences. The medical course is comparable to that of other international universities, with students studying medical information during the first two years and practicing clerkships for the remaining two years.

**Fig. 1:**
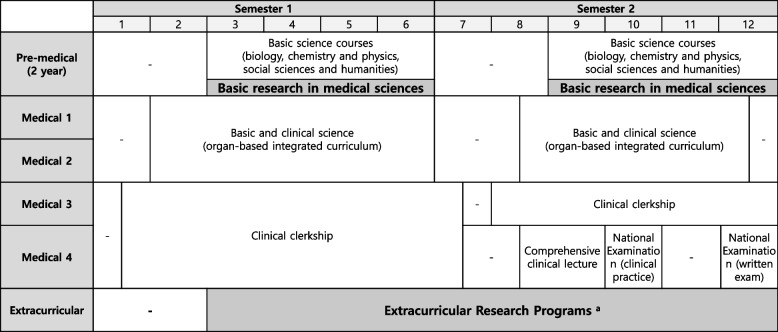
The formal curriculum and Medical Scientist Training Program (MSTP). ^a^ Except for Medical 4

Since the formal curriculum offers students limited research opportunities due to its intense academic workload, HYUCM has run an extracurricular student research program, MSTP, since 2017 (see Fig. 1). Students are not given grades in the program, but they sometimes receive rewards like credits, which the university offers to increase participation and interest [25]. For instance, since 2021, the program has provided one credit. Participating in MSTP is not required for graduation, which means the program is similar to an extracurricular club.

Faculty in the clinical and basic sciences are matched with students to form a research team for two semesters. The institution’s curriculum comprises an initial two years of pre-medical and three to six years of medical school. Sixth-year medical students are excluded from the program because they cannot participate for the entire duration owing to their preparations for clinical practice and the national medical licensing exam. Approximately 10‒20 professor-student teams are created every year. Although the program guarantees operational autonomy for the research teams, there are some guidelines. An interim progress reporting session is scheduled for September and a final result reporting session for December. During the program, students must complete research reports, research notes, and laboratory entry logs, among other documents, which they then submit to the committee after the program ends. The teams can present their results at academic conferences, submit their study to journals, or publish their research; however, these are not mandatory program requirements.

### Participants

Participants were recruited through purposeful sampling which select participants based on specific criteria relevant to the research objective. This approach allowed researchers to gather in-depth and representative data from the population of interest [26]. They were selected based on the following criteria: (1) completed the program at least once, (2) a diversity of years/levels at the university, (3) a diversity of grades earned, and (4) a diversity of research departments (i.e., clinical and basic science). Finally, 18 students (20 cases; 2 students participated twice) participated in the study. Table 1 summarizes the participant characteristics.

**Table 1:** Participant characteristics

Category	*n*
Year	
Pre-medical 1	8
Pre-medical 2	3
Medical 1	-
Medical 2	3
Medical 3	6
Gender	
Female	7
Male	11
Age	
19–21 years	3
22–23 years	5
24–25 years	7
≥ 26 years	5
Faculty department	
Clinical science^a^	15
Basic science^b^	5

^a^Clinical science: Internal Medicine (4); Neurology (1); Orthopedic Surgery (4); Ophthalmology (2); Psychiatry (1); Surgery (1); Urology (2)

^b^Basic science: Microbiology (1); Medical Education (1); Environmental Biology & Medical Parasitology (1); Pathology (1); Biochemistry & Molecular Biology (1)

### Data collection

Data were collected through in-depth, semi-structured interviews from May 2021 to April 2022. Each participant completed a one-on-one interview with the researcher face-to-face. The duration of an interview was one to two hours. The interview questions were developed by incorporating insights from previous research that addressed relevant issues such as how to cultivate undergraduate students' interest in research, and how to sustain that interest over the long-term by implementing effective research programs [6, 8, 10, 17]. The main questions in the interview guide were the following:

- What activities did you usually do? Please tell me about the program process (environment, first meeting with instructors, assignments, etc.).
- What is the most memorable thing for you? Why is it memorable?
- Were there any challenges? If so, how did you feel at the time and how did you overcome it?

To ensure seamless interviews, the main questions were shared with the interviewees in advance. For the interview itself, the researchers prepared probing questions to collect detailed data. Categories were derived from the first 15 interviews. Then, an additional category, called “making a good impression,” was derived after 5 additional interviews were conducted to confirm the saturation of the category. We determined the closure of the interviews based on the point of thematic saturation, which occurs when no further relevant codes or categories can be identified, and recurring issues do not offer additional insights to the study [26–28]. Generally, saturation is achieved through an average of 12–13 interviews for relatively homogeneous samples, and even as few as 9 interviews can be sufficient [27, 28].

### Analysis

The data were analyzed using inductive content analysis, one of the main methods for qualitative research [29, 30]. In this method, cases are collected until they can be categorized as multiple cases of one type, leading to categories based on the units of meaning that are consistent, repeated, and regularly presented. The interviews were recorded with the participants’ consent, and the recorded files were transcribed verbatim. First, each researcher read the transcriptions to fully understand each case. Next, based on the transcribed data, the researchers conducted open coding to list meaningful phrases and describe keywords using the MAXQDA20 (VERBI GmbH, Berlin, Germany, 2019). In this process, coding sheets were produced, and repeated statements or phrases were combined to include specific meanings that make up a group and classified into categories consisting of 3–5 words. For this classification, researchers read the interview transcripts as many times as needed. Finally, abstractions were performed to formulate general explanations through the naming process. A higher-level topic was identified to represent the categories as a similar domain. All these processes were determined after discussions among the researchers.

The researchers discussed the interview data and exchanged feedback regularly (peer debriefing) to enhance the trustworthiness of the study results [31]. Specifically, medical education experts and pedagogy experts reviewed the interpretation of the findings. Additionally, the narratives used for analysis were shared with the students to allow them to verify what they had said. Through this process, the students could check whether their statements were correctly understood and well represented.

## Results

Three domains—learner engagement, instructional design, and program development—and eleven categories were derived from the qualitative analysis. Domain of learner engagement included four subthemes, which were specifically quoted in Table 2. The longitudinal relationship between students and professors has emerged as a crucial factor in terms of instructional design (see Table 3). In every one of the five quotes in Table 4, the complementary relationship between formal curriculum and MSTP was highly emphasized.

**Table 2:** Examples of student comments on learner engagement

Categories	Quote
Perceiving the program as a new experience	I wanted to expose myself to a variety of experiences during the preparatory year. That is why I took part in the conference presentation in addition to doing school activities. (Male, PM1, BS)
	I was looking forward to publishing research papers through this program. The purpose was not for my career. I think research is valuable and awesome because it can change the world. (Male, M3, CS)
Prior experience	The professor told me to write a paper on my own. I had written a paper in the past, and although it was not in the medical field, I thought the description technique would be similar to what I had used back then. So, I wrote it with that past experience in mind. (Male, M2, CS)
	When the research mentor saw me pipetting, he asked me if I had done it before. When I answered that I had, he told me to try it alone after I watched him do the protocols and listen to his explanations. And when he saw me do it, he said, ‘You are following the protocol correctly, so I do not think you need any more explanation.’ (Male, PM1, BS)
Making a good impression	The professor did not pressure me, but I thought I should work harder. If you stay at this university, you have to keep seeing the professor. But I was worried that the professor would think badly of me if I quit the program in the middle, so during the program, I said to myself, ‘Let’s give it a try.’ (Male, M3, CS)
	It was not easy to go out to the lab and do experiments every day because it is vacation time. But I did my best because I am not sure when and where I will meet him [the professor]. (Male, PM1, BS)
Sense of contribution	After seeing how my concerns—like how to use AI when collecting and organizing data, or what research areas could make good use of the data—were reflected in the research, I came to believe that I had developed the mindset of a researcher. (Male, M2, CS)

*PM* Pre-medical, *M* Medical, *CS* Clinical Science, *BS* Basic Science

**Table 3:** Examples of student comments on instructional design

Categories	Quote
Respecting students	The professor did not supervise me a lot, but I was very touched by his warm heart. He always treated me warmly, took good care of me, and answered my questions well. The research process was not easy for me, but I always thought that I should do better because he was a very nice man. (Male, PM2, BS)
Setting clear tasks	We have a journal club every Friday in our lab. The graduate student researcher suggested that everyone ask two questions during every session so that we can all stay focused. I listened carefully during the presentation and tried hard to understand the papers to ask good questions, and then at some point I began to see the flow of the papers. That was interesting. (Female, PM1, CS)
Providing constructive feedback	I tried random assignment using BMI 25, which I learned in class was a standard for obesity. But when my professor heard about it, he gave me feedback, asking whether that method would be appropriate given the distribution of the data I had analyzed so far. After receiving his feedback, I realized I should not take research lightly. (Male, M3, CS)
	I remember the words the professor used to compliment me. He said that I knew more about this topic than he did. This was very inspiring to me, and it motivated me to participate actively in the research. (Male, PM1, CS)
Invitation into the research community	The professor asked other researchers to review my thesis, and they gave very detailed opinions. In particular, I remember that one of them worked very hard during the thesis writing and submission process. After seeing how much they helped me even though they did not know me, I decided that I will also help someone if I find them in a similar situation later on. (Male, PM1, CS)
	At first, I thought the program was being conducted just by the professor. But in the second half, other professors also participated, and I saw that the scale was bigger than I had first imagined. Afterwards, I felt pressured to not make a mistake. (Male, PM1, BS)

*PM* Pre-medical, *M* Medical, *CS* Clinical Science, *BS* Basic Science

**Table 4:** Examples of student comments on program development

Categories	Quote
Setting the program assignment	As I consistently recorded my research notes, I could see where I lacked an understanding of the research flow. I was able to quickly make up for the shortcomings of the research. (Female, M2, CS)
	Through the presentations in reporting sessions, I had the opportunity to see how and what progress was being made in other students’ research. It felt like a time trial, and I was under pressure to do my research as quickly as possible. I think it was a tool used for producing the final results. (Female, M2, CS)
Developing mutual support between the formal curriculum and MSTP	I was able to find references more easily this year. It was hard last year because I had never presented a journal article. But this year, I found more references and did better research than I did last year. (Female, M3, CS)
	Because the clerkship time was fixed, the most difficult part was arranging the schedule with the professor. Furthermore, the hospital where the professor works and the hospital where I practice are not the same. As a result, there was little communication. (Male, M3, CS)
Securing time to spare	In terms of time, I did not have any conflicts while I participated in the program. This is because the school lectures were recorded and shared online due to the COVID-19 pandemic. I could replay any portions that I did not initially understand. Consequently, my overall study time was shorter, and I could spend more time participating in research activities. (Male, M2, CS)

*MSTP* Medical Scientist Training Program, *PM* Pre-medical, *M* Medical, *CS* Clinical Science, *BS* Basic Science

### Domain of learner engagement

#### Perceiving the program as a new experience

The desire for new experiences led students to participate in the research program. Students reported that they felt “bored with the same curriculum,” which is focused on conveying medical knowledge, and they expected to have diverse experiences in MSTP. They recognized the program as an opportunity to “officially escape from studying,” “a new stimulation,” and “vitality for their daily routine.” To meet these expectations, they accepted new challenges, such as using an artificial intelligence program, cell experiments, animal experiments, and conference presentations. Most students said that the program was valuable because they could participate in research activities, regarding acquiring authorship as a byproduct of the experience. Some students expected authorship; however, most students valued publishing experience without it serving for career advancement.

#### Prior experience

Prior research experience had a positive effect on students’ ability to perform a task without supervision and work at a higher level. The students’ prior experiences consisted of scientific writing, using statistics programs, and animal testing or experiments on cells. These experiences helped the students perform their tasks even if the supervisor did not demonstrate the method. Some skills that the students learned (e.g., coding and meta-analysis) lessened or removed the need for the basic learning stage and raised the expectations of team members, including the supervisor. This allowed the students to experiment independently and accelerated the research by engaging them directly in practice roles such as data collection.

#### Making a good impression

The desire to make a good impression prompted the students to strive to produce the best results possible. Students who wanted to build a good student-faculty relationship pushed themselves to not disappoint their supervisors. For example, they tried to increase the task quality by devoting extra time and effort searching for materials and studying. Regardless of whether the research was difficult or different from their expectations, they did not give up.

#### Sense of contribution

A sense of contribution increased the students’ satisfaction and confidence about the research and promoted their research engagement. Most of the students thought that they contributed to the research, such as reducing the time needed for data collection or refining research methods. In this process, the students developed their interests and formed a researcher’s mindset.

### Domain of instructional design

#### Respecting students

A supervisor’s respect for the students was one of the key factors that motivated the students to participate enthusiastically in the research program and encouraged them to advance their ideas. Most of the students were involved in ongoing research and reported that they felt “nervous” and “found it difficult to express their ideas or ask questions.” However, when the supervisor answered all their questions, coordinated meeting times, allowed them autonomy to choose a task rather than directing them, and accepted their opinions, they realized that their supervisor respected them. In response, they dedicated their best to the program and actively communicated their ideas.

#### Setting clear tasks

A clear task is specific in content, deadline, method, and purpose. It provides the students with immediate goals that they might achieve in a short amount of time, in the context of long-term research. This includes attending research meetings or journal clubs and participating in brief quizzes. By achieving short-term goals, the students gained “value” and “new insights” into the research, which stimulated their research interest and confidence. The students completed tasks with greater ease by referring to a bibliography or prior research proposal provided by their supervisor. Additionally, when the supervisor explained the intention or value of the task or program, the students tried to comply.

#### Providing constructive feedback

Constructive feedback, which is both positive and corrective, improved the students’ perception of research. Positive feedback increased the students’ confidence by making them feel recognized for their efforts. It included direct comments, such as compliments on the students’ competency, and indirect comments, such as suggestions for authorship. The students regarded this as “proof of proper research execution” and “motivation to continue research.” Moreover, corrective feedback aided in setting the direction for research and provided students the opportunity to grow. Receiving feedback on ideas or assignments helped the students modify their thoughts and produce better outputs, and it provided them an opportunity to think critically about the research.

#### Invitation into the research community

Inviting students into the research community increased the scope and depth of their learning. The community offered students the opportunity to observe the process of communicating among researchers from different academic backgrounds. They also learned about diverse perspectives on the research theme and acquired and internalized the researchers’ attitudes and postures. Moreover, the community provided learners with the option to observe the process of research development. Although this increased the pressure on students by giving them the impression that the research was becoming more intense, it also served as positive pressure that encouraged them to perform their duties meticulously, resulting in greater depth of the research.

### Domain of program development

#### Setting the program assignment

Assignments given to the students included research notes, reports, and presentations. Writing research notes helped the students improve their understanding of the data. While taking notes, they reflected on the research process and filled in any gaps that they discovered. Reports and presentations helped organize the research process and accelerate its progress. However, the students felt nervous about their work when they compared their results with those of the other students during the presentation.

#### Developing mutual support between the formal curriculum and MSTP

While learning the formal curriculum gave the students confidence to proceed in the research program, their general lack of knowledge contributed to their anxiety. The knowledge gained through the curriculum contributed to alleviating such anxiety. Some students used the statistical knowledge they had gained from statistics lectures to analyze data, whereas others applied what they had learned from other lectures to perform experiments. Additionally, problem-based learning and journal presentations, taught as part of the formal curriculum, helped the students become more comfortable with acquiring references and participating in MSTP research.

#### Securing time to spare

Having spare time enables students to participate in research programs. For example, the summer vacation is a critical period for both pre-medical and medical students, especially for those who plan to make progress through intensive research activities. Students also have more free time in the pre-medical phase than in the medical phase, which allows them to meet with their supervisors frequently. During the medical phase, clerkship schedules had to be less demanding so that the students can engage in intensive research. Additionally, the use of online classes, owing to the spread of COVID-19, facilitated the students’ participation in the research program. As the space–time constraints of the classroom environment disappeared, students integrated lectures and reviews instead of considering them separately.

### Discussions

The findings of this study provide important insights into how medical students can effectively acquire research capabilities in a short period of time through participation in a research program, with the results divided into three domains: learner engagement, instructional design, and program development. Students at HYUCM described their thoughts and experiences on participating in MSTP in addition to the formal curriculum despite their busy school schedules. Thus, this study offers a learner-centered view of medical students’ engagement in research.

Since medical education in Korea is currently shifting toward a learner-centered as opposed to instructor-centered approach, recognizing this is an important step in improving student engagement in the research program. Our findings demonstrate that the students successfully performed tasks using their prior research experience and their performance could improve their research confidence leading to active engagement. Interestingly, all the participants except one had research experience. Previous studies have noted the importance of prior experience, which can increase research interest and promote participation [6, 20, 32]. Prior experience reduced the psychological barrier to starting research and helped solve problems encountered in the research process. It is also important to make students feel that they have contributed to research. A sense of contribution to the research enhanced research engagement by increasing students’ self-satisfaction and confidence in the research capacity [33]. Further, students who felt that they made substantial contributions to the research actively participated, took initiative, and formed a researcher mindset [12]. Through this process, students usually acquired in-depth knowledge and research skills, and our participants also could learn how to think and communicate like a researcher [34].

It was found that the role of a supervisor was also crucial for students to take the initiative in participating in the research program. Consistent with the literature, our study found that the research supervisor played a crucial role in enhancing research engagement, which creates an environment that fosters students’ enthusiasm and intrinsic motivation [35]. The emphasis in our study is that it is important for supervision to be carried out with the students’ position in mind. Students’ autonomy was maintained by supervisors “respecting students.” Several students mentioned that they initially hesitated to present their opinions; however, when their supervisor listened to them attentively, they became “active” in the research. These findings indicate that assisting students’ learning and allowing them to take ownership of the research is a suitable methodology for improving program quality. Also, students’ competence could be boosted by the supervisor “setting clear tasks” and “providing constructive feedback.” Through the task-solving process, students acquired confidence in their abilities, which increased their interest in science and specific research areas. In particular, the assigned tasks were often attainable in the short-term even at the student level, though the research was lengthy. In accordance with these results, promoting success via easily attainable tasks was a strategy used to enhance research self-efficacy and interest [36, 37].

The research program must be in line with making students concentrate on research and exercise their capabilities to strengthen students’ research motivation and increase the probability of their participation. Specifically, the formal curriculum should allow time for students to focus on research, while MSTP should complement the formal curriculum by targeting program outcomes. To successfully complete the program assignments, the students had to meticulously acquaint themselves with the research process. In particular, the research presentation encouraged self-reflection through exchanging peers’ task reviews. It has been shown that authentic research tasks, such as writing a research report and research presentations, foster students’ intrinsic motivation for research [38]. These requirements urged students to review the research process regularly and set up improvement plans on their own.

This study has several interesting findings distinguished from those of previous studies. First, the student-faculty relationship is newly discovered as a factor influencing student research engagement and is closely relevant to the Korean context. People usually manage the impressions they make to create and maintain the images they want. The students in our study also attempted to make a good impression to faculty, and this positively affected their research engagement. Students' academic success or sense of belonging is greatly influenced by the supervisor's belief in them, and specifically, they have determined their behavior by focusing on regular social interactions so as not to disappoint others [39, 40].

Furthermore, most mentor–mentee relationships in medical schools are longitudinal in Korea. They have a teacher-student relationship at school and then work together in the same hospital after graduation; thus, the network established between students and instructors is expected to continue for a long period [41]. These human characteristics and the context of the student-professor longitudinal relationship in medical schools are also reflected in our study. Since over half of HYUCM students train and continue their careers in the same educational hospitals, there is a high likelihood that students and faculty members will work together in the future. Students in this organizational environment pay great attention to their reputation management reflected in the supervisor. As a result, the students heavily invested their time and resources to show the best results to their supervisor in the program. Therefore, it can be inferred that the students were conscious that their relationship with their professors would affect their college life and their future career, and they self-promoted as a strategy to leave a good impression [42].

On the other hand, inviting students into the supervisors’ research community had mixed impacts on the students’ research engagement. The students gained new insights into their research and learned about researcher interactions through discussions with community members. Seeing the extended research community allowed them to grasp the importance and intensity of the work. This served as a positive pressure that encouraged them to conduct research meticulously. However, the academic networking caused some students to feel stressed, contrary to the findings of previous studies that communities benefit students by allowing them to learn collaborative research skills. This contradiction may be because the people students met were colleagues of their teachers or researchers rather than their peers. The students may have been more sensitive to their relationships with faculty due to the authoritarian culture of Korean medical schools [43]. Since the faculty-student hierarchy is a typical characteristic of college cultures [44, 45], the gap between the status of the faculty and students might discourage students from being involved more positively in research process.

Next, the study participants actively engaged in the research program despite the COVID-19 pandemic. In previous studies, it has been reported that students’ lack of time has consistently been cited as a significant research barrier, even up to the present [9, 18, 21, 35]. However, in this study, pre-clerkship students preferred to participate in the research program because their courses had shifted to the online mode under the pandemic. They were able to make efficient use of learning time to shorten the period of review by using the pause/play and speed options, in addition to applying flexible scheduling to view learning videos [46]. Thus, COVID-19 and the transition to online learning could afford students more time available for extracurricular research activities.

We were able to identify the research training program’s advantages by focusing on students who participated in MSTP while completing the formal curriculum. The students who desired a new experience felt that the research experience was valuable. This is because the MSTP activity has traits that can supplement the limitations of the formal curriculum while also providing a variety of learner-centered experiences [47]. A previous study found that publication rewards were a strong motivator for medical students [11, 48]. Conversely, we noted that the participants recognized and valued the research program as an opportunity for new experiences and distinguished it from the simple acquisition of medical knowledge. The selection and matching process ensured that the students in MSTP were motivated to see the research opportunity as a privilege, take advantage of the scientific environment, and actively conduct research [6]. These motivations were characteristics of both students and supervisors who volunteered in the program [45, 49].

The results of this study suggest that the following should be considered when implementing a research program to promote students’ research capabilities. First, the program director or committee can consider offering faculty development opportunities to help professors design learner-centered instructional design. To strengthen students’ intrinsic motivation, it is necessary to know and meet their needs and motivational points. Encouraging supervisors as learner-centered mentors is also expected to be effective in creating a good environment for students to strengthen their research capabilities [8]. Secondly, it is critical for faculty members to create a comfortable atmosphere in the research program to encourage student participation. If there is a prominent hierarchical relationship, students may feel hesitant to contribute. To address this issue, faculty can implement strategies such as carefully selecting their words and continuously encouraging students [40]. Additionally, demonstrating sympathy and respect towards students can effectively control negative emotions and enhance participation [50]. Last but not least, providing an orientation that introduces students to how to operate a research program is necessary. It is advisable to clarify the value of the role assigned to students, no matter how small, and provide them with the opportunity to work autonomously to create a sense of belonging within the research team's cooperative structure [51]. Providing supervisors with various tips for program operation can also increase the effectiveness of research programs.

This study needs some cautions to be considered. Although qualitative studies can provide more detailed information to explain the complex issues related to individual experiences, the single-center investigation limits generalizability to other programs and settings. Investigating other university programs with the aim of improving student research engagement will be useful [17]. Second, since the study participants included those who volunteered to be a part of the research training program, their motivation may have been higher from the beginning; therefore, our sample may not be representative of all medical students. Lastly, the findings of this study are based solely on the perceptions of the students and do not include all stakeholders involved in the program. To gain a more comprehensive understanding of the facilitators of students’ research engagement, together with students’ perceptions, the viewpoints of other stakeholders of the program must be investigated.

## Conclusions

This study presents the perspectives of medical students to shed light on the context in which their research engagement is promoted and what supports are beneficial, from a learner-centered view. The importance of considering both instructional design and program development from the perspective of students is emphasized. The longitudinal relationship between students and professors has newly emerged in the Korean context as a factor that strengthens student engagement in research. The students were conscious that their relationship with their professors would affect their college lives as well as their future careers, and they self-promoted as a strategy to leave a good impression. The complementary relationship between formal curriculum and MSTP has been also highlighted to encourage student engagement in research.

## Data Availability

The datasets of this article are available from the corresponding author on reasonable request.

## Abbreviations

MSTP: Medical Scientist Training Program
AI: Artificial Intelligence
BS: Basic Science
CS: Clinical Science
HYUCM: Hanyang University College of Medicine

## References

[1] Chang Y, Ramnanan CJ (2015). A review of literature on medical students and scholarly research: experiences, attitudes, and outcomes. Acad Med.

[2] Möller R, Shoshan M, Heikkilä K (2015). What is the reward? Medical students’ learning and personal development during a research project course. Med Educ Online.

[3] Tae Y-H, Park S-K, Cho Y-R. A basic study for monitoring Physician Workforce activity patterns. Seoul: National Health Insurance Service; 2020.

[4] Song WJ, Lee S-H, Chung JH (2022). Current status and future direction of physician-scientists training in Korea. JID Innovations.

[5] Frishman WH (2001). Student research projects and theses: should they be a requirement for medical school graduation?. Heart Dis (Hagerstown, Md).

[6] Öcek Z, Batı H, Sezer ED (2021). Research training program in a Turkish medical school: challenges, barriers and opportunities from the perspectives of the students and faculty members. BMC Med Educ.

[7] Laidlaw A, Aiton J, Struthers J, Guild S (2012). Developing research skills in medical students: AMEE Guide No. 69. Medical Teach.

[8] Möller R, Wallberg A, Shoshan M (2021). Faculty perceptions of factors that indicate successful educational outcomes of medical students’ research projects: a focus group study. BMC Med Educ.

[9] Murdoch-Eaton D, Drewery S, Elton S (2010). What do medical students understand by research and research skills? Identifying research opportunities within undergraduate projects. Med Teach.

[10] Ommering BW, van Blankenstein FM, Waaijer CJ, Dekker FW (2018). Future physician-scientists: could we catch them young? Factors influencing intrinsic and extrinsic motivation for research among first-year medical students. Perspect Medical Educ.

[11] Ommering BW, Wijnen-Meijer M, Dolmans DH, Dekker FW, van Blankenstein FM (2020). Promoting positive perceptions of and motivation for research among undergraduate medical students to stimulate future research involvement: a grounded theory study. BMC Med Educ.

[12] Solomon SS, Tom SC, Pichert J, Wasserman D, Powers AC (2003). Impact of medical student research in the development of physician-scientists. J Investig Med.

[13] Barron JS, Bragg E, Cayea D, Durso SC, Fedarko NS (2015). The short-term and long-term impact of a brief aging research training program for medical students. Gerontol Geriatr Educ.

[14] Jacobsen GW, Ræder H, Stien MH, Munthe LA, Skogen V (2018). Springboard to an academic career—A national medical student research program. PLoS One.

[15] Devi V, Ramnarayan K, Abraham RR, Pallath V, Kamath A, Kodidela S (2015). Short-term outcomes of a program developed to inculcate research essentials in undergraduate medical students. J Postgrad Med.

[16] Yune S-J, Park Y-S, Cho J-H (2018). Factors That Influence Educational Effectiveness and Learning Satisfaction in Biomedical Research Programs during Premedical School. Korean Med Educ Rev.

[17] Mugabo E, Velin L, Nduwayezu R (2021). Exploring factors associated with research involvement of undergraduate students at the College of Medicine and Health Sciences University of Rwanda. BMC Med Educ.

[18] El Achi D, Al Hakim L, Makki M (2020). Perception, attitude, practice and barriers towards medical research among undergraduate students. BMC Med Educ.

[19] Burgoyne LN, O'Flynn S, Boylan GB (2010). Undergraduate medical research: the student perspective. Med Educ Online.

[20] Rosenkranz SK, Wang S, Hu W (2015). Motivating medical students to do research: a mixed methods study using Self-Determination Theory. BMC Med Educ.

[21] de Oliveira NA, Luz MR, Saraiva RM, Alves LA (2011). Student views of research training programmes in medical schools. Med Educ.

[22] Muhandiramge J, Vu T, Wallace MJ, Segelov E (2021). The experiences, attitudes and understanding of research amongst medical students at an Australian medical school. BMC Med Educ.

[23] Wahlqvist M, Skott A, Björkelund C, Dahlgren G, Lonka K, Mattsson B (2006). Impact of medical students' descriptive evaluations on long-term course development. BMC Med Educ.

[24] Burk-Rafel J, Harris KB, Heath J, Milliron A, Savage DJ, Skochelak SE (2020). Students as catalysts for curricular innovation: a change management framework. Med Teach.

[25] Han A, Kwon K (2018). Students' Perception of extracurricular activities: a case study. J Adv Educ Res.

[26] Seidman I. Interviewing as qualitative research: a guide for researchers in education and the social sciences. 3rd ed. New York: Teachers College Press; 2006.

[27] Hennink MM, Kaiser BN, Marconi VC (2017). Code saturation versus meaning saturation: how many interviews are enough?. Qual Health Res.

[28] Hennink M, Kaiser BN (2022). Sample sizes for saturation in qualitative research: a systematic review of empirical tests. Soc Sci Med.

[29] Tavakol M, Sandars J (2014). Quantitative and qualitative methods in medical education research: AMEE Guide No 90: Part II. Med Teach.

[30] Elo S, Kyngäs H (2008). The qualitative content analysis process. J Adv Nurs.

[31] Lincoln YS, Guba EG. Naturalistic inquiry. Newbury Park: Sage Publications; 1985.

[32] Siemens DR, Punnen S, Wong J, Kanji N (2010). A survey on the attitudes towards research in medical school. BMC Med Educ.

[33] Ommering BW, van Diepen M, van Blankenstein FM, de Jong PG, Dekker FW (2020). Twelve tips to offer a short authentic and experiential individual research opportunity to a large group of undergraduate students. Med Teach.

[34] Canfield J, Truong V, Bereznicka A (2023). Evaluation of a student clinical research education program in addiction medicine. Ann Med.

[35] Murray H, Payandeh J, Walker M. Scoping Review: Research Training During Medical School. Med Sci Educ. 2022;32(6):1553-61. 10.1007/s40670-022-01679-7 PMC9755431 36532387

[36] Robnett RD, Chemers MM, Zurbriggen EL (2015). Longitudinal associations among undergraduates' research experience, self-efficacy, and identity. J Res Sci Teach.

[37] Bandura A. Self-Efficacy: The Exercise of Control. New York: Worth Publisher; 1997.

[38] Ommering BW, van Blankenstein FM, van Diepen M, Dekker FW (2021). Academic success experiences: promoting research motivation andself-efficacy beliefs among medical students. Teach Learn Med.

[39] Goffman E (2021). The presentation of self in everyday life Anchor.

[40] Lytle A, Shin JEL (2023). Self and professors’ incremental beliefs as predictors of STEM engagement among undergraduate students. Int J Sci Math Educ.

[41] Dimitriadis K, von der Borch P, Störmann S (2012). Characteristics of mentoring relationships formed by medical students and faculty. Med Educ Online.

[42] Jones EE, Pittman TS (1982). Toward a general theory of strategic self-presentation. Psychol Perspect Self.

[43] Chun K, Park W, Lee S, Park Y, Kang E (2010). A study on the educational climate, self-directed learning and creative thinking in medical school. Korean J Think Dev.

[44] Knight SE, Van Wyk JM, Mahomed S (2016). Teaching research: a programme to develop research capacity in undergraduate medical students at the University of KwaZulu-Natal. South Afr BMC Med Educ.

[45] Ho T, Agarwal A, Khambhati J, Sarfaty S, Hirsch AE (2017). Faculty and student evaluations of a medical student summer research program: a 15 year analysis. Med Sci Educ.

[46] Kang Y, Kim D-H (2021). A qualitative study on the perceptions and learning behavior of medical students in online classes. Korean Med Educ Rev.

[47] Lunenburg FC (2010). Extracurricular activities. Schooling.

[48] Gnjidic D, da Costa N, Wheate NJ (2023). Potential factors that can affect the performance of undergraduate pharmacy research students: a descriptive study. BMC Med Educ.

[49] Ommering BW, van den Elsen PJ, van der Zee J, Jost CR, Dekker FW (2018). Using an extracurricular Honors program to engage future physicians into scientific research in early stages of medical training. Med Sci Educ.

[50] Hsu JL, Goldsmith GR (2021). Instructor strategies to alleviate stress and anxiety among college and university STEM students. CBE-Life Sci Educ.

[51] Hughes AM, Salas E (2013). Hierarchical medical teams and the science of teamwork. AMA J Ethics.

